# The unseen struggle—depression and associated factors in geriatric cancer patients

**DOI:** 10.3389/fmed.2025.1603515

**Published:** 2025-07-24

**Authors:** Ozlem Dogan, Hayriye Sahinli, Dogan Yazilitas, Selen Kantarci

**Affiliations:** ^1^Department of Medical Oncology, Adiyaman University Training and Research Hospital, Adıyaman, Türkiye; ^2^Department of Medical Oncology, Ankara Etlik City Hospital, Ankara, Türkiye

**Keywords:** cancer, depression, older adult, geriatrics, insomnia

## Abstract

**Background:**

The objective of this study was to investigate the frequency of depression and its associations, rather than causal relationships, in patients aged 65 years and older receiving chemotherapy, using the Geriatric Depression Scale (GDS).

**Methods:**

This prospective study was conducted between January 2023 and December 2023 at Ankara Etlik City Hospital, including 501 chemotherapy patients aged 65 years and older. Patients receiving only oral therapy, those under palliative care, those with brain metastases, or those with insufficient cognitive functionality were excluded. Demographic and clinical data were collected from medical records. Depression was assessed using the 15-item Yesavage Geriatric Depression Scale (GDS), with scores ≥5 indicating high depression symptoms.

**Results:**

Among the 501 patients included in the study, 204 (40.7%) were female, with a median age of 69 years (range: 65–84 years). A total of 214 patients (42.7%) had high depressive symptom scores (GDS ≥ 5). A multivariable logistic regression analysis identified the following as independent predictors of depression: being female (odds ratio (OR): 1.481, 95% confidence interval (CI): 1.011–2.168, *p* = 0.04), body mass index (BMI) ≥ 21 (OR: 1.665, 95% CI: 1.081–2.564, *p* = 0.02), higher pain scores (OR: 1.269, 95% CI: 1.122–1.436, *p* < 0.001), insomnia (OR: 1.626, 95% CI: 1.109–2.384, *p* = 0.01), and weak social support (OR: 2.004, 95% CI: 1.046–3.839, *p* = 0.03).

**Conclusion:**

Our study highlights the high prevalence of depressive symptoms among geriatric cancer patients. In this population, early diagnosis and management of depression, with particular attention to independent risk factors such as pain and insomnia, as well as strengthening social support mechanisms, may be crucial for enhancing quality of life and improving treatment adherence.

## Introduction

Advancements in healthcare systems and medical technologies have significantly increased the proportion of older adult populations worldwide. Given that the incidence of cancer increases with age, approximately 60% of newly diagnosed cancer patients are older adults (1). Patients aged 65 years and older undergoing cancer treatment not only face the physical challenges of therapy but also endure substantial psychological stress. Depression is widespread in this group, yet symptoms are often overlooked, leading to underdiagnosis (2). This oversight can negatively impact quality of life, reduce adherence to treatment, and ultimately compromise overall survival outcomes (3).

The development or progression of new comorbidities, as well as the loss of spouses and friends, makes older adult patients more vulnerable to depression (4). The stress associated with a cancer diagnosis and its treatment further exacerbates this risk. Due to its potential presentation with atypical symptoms, depression in older adults can be challenging to diagnose and treat. When left untreated, it may worsen physical symptoms and deteriorate overall health status. Therefore, timely diagnosis and effective management of depression are particularly critical in older adult cancer patients (2–5).

The Yesavage Geriatric Depression Scale (GDS) is a widely used, specialized tool developed to screen for depression in the geriatric population. This tool’s simple and comprehensible structure allows for the effective identification of depressive symptoms even in patients with physical and cognitive limitations. It is an ideal instrument for recognizing depression in older adult patients undergoing intensive treatments such as chemotherapy (6–9).

Our study aims to evaluate the prevalence of depression and identify associated factors in patients aged 65 years and older undergoing chemotherapy, utilizing the GDS.

## Materials and methods

Between January 2023 and December 2023, our prospective study was conducted at Ankara Etlik City Hospital and included 501 patients aged 65 years and older who were receiving chemotherapy in the outpatient treatment unit and provided written informed consent to participate. A total of 601 patients were initially screened for eligibility. Patients receiving oral therapy only (*n* = 58), those under palliative care (*n* = 32), those with known brain metastases (*n* = 4), and those with insufficient cognitive functionality to complete the survey (*n* = 6) were excluded.

**Figure 1:**
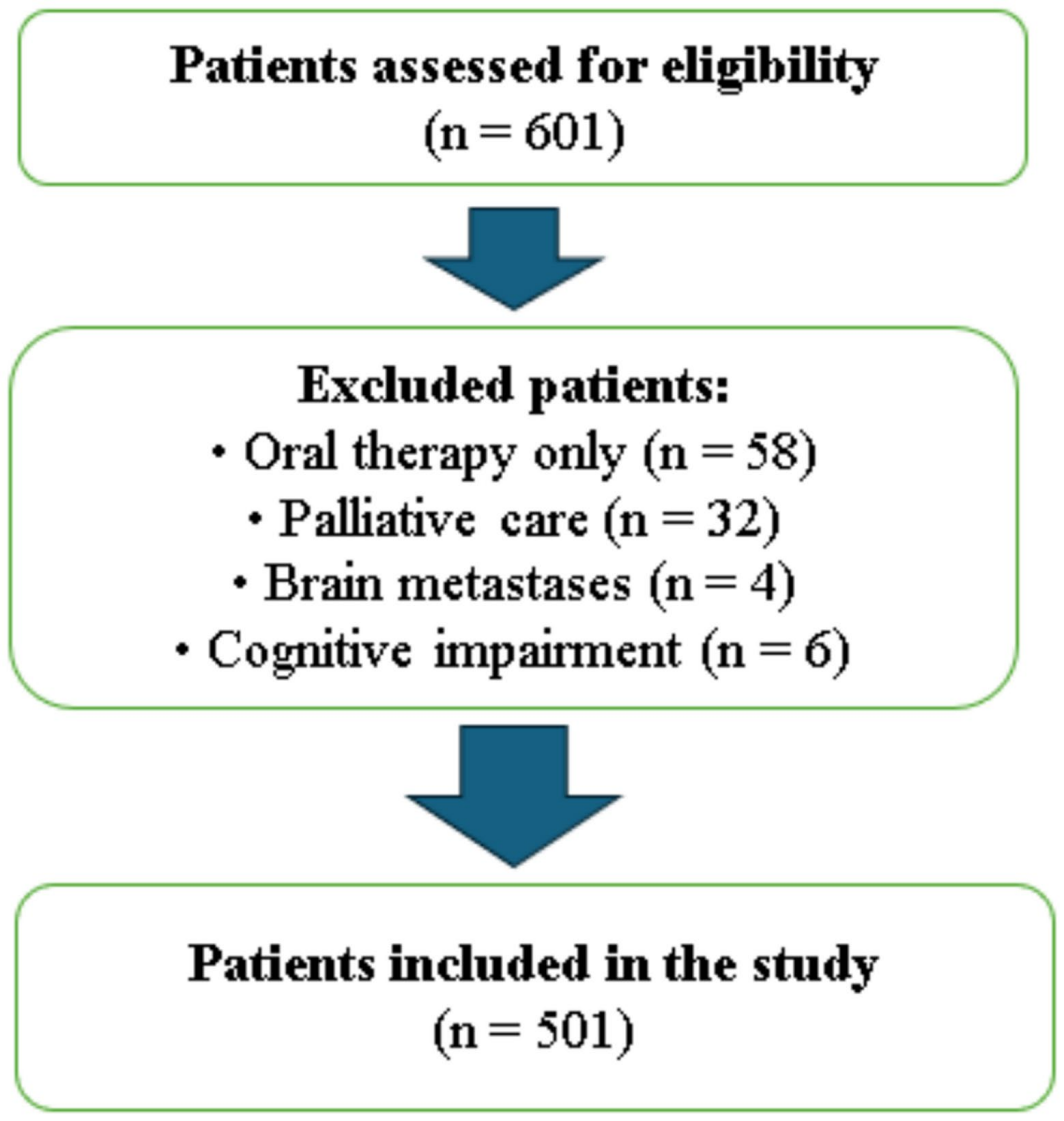
Flow diagram of patient selection and inclusion in the study.

Patients with cognitive impairment were excluded based on the attending oncologist’s clinical judgment, who assessed the patients’ ability to understand and respond to the questionnaires during pre-chemotherapy evaluations. No standardized cognitive screening tool was used. Additionally, all patients with known brain metastases were excluded, regardless of whether they were symptomatic or asymptomatic, to minimize potential confounding effects on cognitive and psychological assessments. After applying these criteria, 501 eligible patients were included and completed all study procedures (Figure 1).

Demographic and clinicopathological characteristics of the patients were obtained from the hospital’s electronic medical records and patient files. The recorded data included age, sex, Eastern Cooperative Oncology Group (ECOG) performance status, BMI, comorbidities, history of prior depression and antidepressant use, alcohol consumption, educational and occupational background, type and stage of cancer, history of radiotherapy or surgery, awareness of diagnosis, and time since initiation of cancer treatment. The choice of BMI < 21 as a threshold for poor nutritional status was based on the American Society of Clinical Oncology (ASCO) guideline for geriatric oncology, which recommends nutritional risk screening in older adult patients (10). Unlike the World Health Organization (WHO) classification designed for the general population, this threshold is specifically tailored for older adults and reflects geriatric vulnerability more accurately (11).

To assess the level of social support, patients were categorized based on their living conditions as either living with family members (strong social support) or living alone, with a caregiver, or in a nursing home (weak social support). Pain status was evaluated using the Numeric Rating Scale (NRS), where patients rated their pain on a scale of 0–10 (12). The study was conducted in accordance with the principles of the Declaration of Helsinki and was approved by the Ethics Committee of Dışkapi Yildirim Beyazit Research and Training Hospital.

### Assessment of depression

All patients were administered the 15-item short form of the Yesavage Geriatric Depression Scale (GDS), which has been validated for use in Türkiye and adapted for use in the Turkish language. The GDS is a specialized tool with responses recorded as “yes” or “no.” It is simple and easy to understand, making it suitable for use in geriatric patients and those with physical or cognitive limitations (7–9).

Patients completed the questionnaires during their chemotherapy sessions while seated and in a resting state, typically at the beginning or midway through the infusion process. This timing was chosen to avoid fatigue or discomfort that may arise near the end of treatment. The environment was kept calm and standardized across all sessions to ensure consistency. When needed, caregivers or healthcare personnel provided assistance to help patients understand or complete the items. A score of 0–4 on the GDS was categorized as indicating low depressive symptoms, while a score of ≥5 was classified as high depressive symptoms. All enrolled patients completed the GDS in full, with no missing data for this variable.

### Assessment of insomnia

The Insomnia Severity Index (ISI) was used to assess insomnia. The ISI is a seven-item scale. Responses are scored on a scale of 0–4. Scores of 0–7 indicate clinically insignificant insomnia, 8–14 indicate mild insomnia, 15–21 indicate moderate insomnia, and 22–28 indicate severe insomnia (13). This scale was administered to patients alongside the GDS during their chemotherapy sessions. All enrolled patients completed the ISI in full, with no missing data for this variable.

### Statistical analysis

Continuous variables were expressed as median (minimum–maximum). The conformity of continuous variables to a normal distribution was analyzed using the Kolmogorov–Smirnov test. Continuous variables with a normal distribution were analyzed using the independent samples *t*-test, while those without a normal distribution were analyzed using the Mann–Whitney U test. The relationship between clinicopathological characteristics and depression scale scores was analyzed using the *χ*^2^ test. Results with a *p*-value of <0.05 were considered statistically significant. Variables identified as statistically significant in the univariate analysis were included in a multivariable logistic regression model to determine independent predictors. Additionally, an *a priori* power analysis determined that a minimum sample size of 500 patients was required to detect statistically significant differences with 80% power at a 0.05 significance level. All statistical analyses were performed using Statistical Package for the Social Sciences (SPSS) version 21.0 (SPSS, Inc., Chicago, IL, USA).

## Results

Among the 501 patients included in the study, 204 (40.7%) were female, and 297 (59.3%) were male. The median age was 69 years (range: 65–84 years). The ECOG performance score was 0 in 106 patients (21.2%), while 395 patients (78.8%) had a score of 1–2. A total of 455 patients (90.8%) lived with their families. Regarding education, 373 patients (74.5%) had completed primary school, 53 (10.6%) completed high school, 28 (5.6%) were university graduates, and 47 (9.4%) had no formal education. A total of 272 patients (54.3%) had no comorbidities, and 460 patients (91.8%) were not employed. Two patients (0.4%) had a prior diagnosis of depression, while 499 (99.6%) had no such history. Seven patients (1.4%) reported a history of antidepressant use. Among the 204 female patients, 170 (83.3%) were homemakers. Among the 297 male patients, 38 (12.8%) were actively employed before their diagnosis, compared to only 3 female patients (1.5%). Of the patients, 371 (74.1%) had never consumed alcohol, while 96 (19.2%) had consumed alcohol at some point in their lives but had quit. The majority of patients (*n* = 424, 84.6%) were aware of their cancer diagnosis, while a small number (*n* = 27, 5.4%) were unaware of their diagnosis. For 219 patients (43.7%), the duration since chemotherapy initiation was 6 months or longer. Regarding cancer types, 225 patients (44.99%) had gastrointestinal cancers, 99 (19.8%) had lung cancer, and 66 (13.2%) had breast cancer. A total of 251 patients (50.1%) had stage 2–3 disease, while 250 (49.9%) were diagnosed with stage 4 cancer. Surgical history was present in 217 patients (43.3%), and 120 patients (24%) had undergone radiotherapy (RT). Additionally, 379 patients (75.6%) had a body mass index (BMI) of ≥21. The clinicopathological and demographic characteristics of the patients are summarized in Table 1.

**Table 1:** Patient characteristics (*n* = 501).

Variables	*n* (%)	Variables	*n* (%)
Sex		Time since cancer diagnosis	
Female	204 (40.7)	<6 months	282 (56.3)
Male	297 (59.3)	≥6 months	219 (43.7)
ECOG		Stage	
0	106 (21.2)	Stages 2–3	258 (50,1)
1–2	395 (78.8)	Stage 4	250 (49.9)
Educational background		Awareness of disease	
No education	47 (9.4)	Aware	424 (84.6)
Primary school	373 (74.5)	Partially aware	50 (10.0)
High school	53 (10.6)	Unaware	27 (5.4)
Undergraduate/graduate	28 (5.6)	Operated due to cancer	
Employment status		Yes	217 (43.3)
Yes	41 (9,2)	Type of cancer	
Number of comorbidities		Breast	66 (13.2)
1	127 (25.3)	Gastrointestinal	225 (44.9)
2	69 (13.8)	Lung	99 (19.8)
3	40 (6)	Gynecologic	38 (7.6)
4	3 (0.6)	Genitourinary	17 (3.4)
Social support		Other	56 (11.2)
Strong (living with family)	455 (90.8)	Depression scale	
Weak (lives alone/with caregiver/at nursing home)	46 (9.2)	≥5	214 (42.7)
Use of alcohol		0–4	287 (57.3)
Yes	34 (6.8)	Use of antidepressants	
Diagnosed with depression		Yes	7 (1.4)
Yes	2 (0.4)	Radiotherapy	
		Yes	120 (24.0)

According to the Geriatric Depression Scale (GDS), 214 out of 501 patients (42.7%) had high depressive symptom scores (GDS ≥ 5). In the univariate analysis, high depressive symptoms were significantly associated with being female (*p* = 0.04), weak social support (*p* = 0.009), BMI ≥ 21 (*p* = 0.01), longer treatment duration (≥6 months) (*p* = 0.03), higher pain scores (*p* < 0.001), and insomnia scores (*p* = 0.01). No statistically significant associations were found with ECOG performance status (*p* = 0.05), alcohol use (*p* = 0.59), educational level (*p* = 0.09), occupational status (*p* = 0.24), cancer stage (*p* = 0.82), or cancer type (*p* = 0.14) (Table 2).

**Table 2:** Comparison of patient characteristics with the Geriatric Depression Scale.

Variables *n* (%)	Low depressive symptom score *n* = 287	High depressive symptom score *n* = 214	*p*-value*
ECOG			
0	52 (18.1)	54 (25.2)	0.05
1–2	235 (81.9)	160 (74.8)	
Awareness of disease			
Aware	242 (84.3)	182 (85.0)	0.82
Partially aware/unaware	45 (15.7)	32 (15.0)	
Sex			
Female	106 (36.9)	98 (45.8)	0.04
Male	181 (63.1)	116 (54.2)	
Educational background			
No education	20 (7.0)	27 (12.6)	
Primary school	220 (76.7)	153 (71.5)	0.09
High school/Undergraduate/graduate	47 (16.4)	34 (15.9)	
Social support			
Strong (living with family)	269(93.7)	186(86.9)	
Weak (lives alone/with caregiver/ at nursing home)	18(6.3)	28(13.1)	0.009
Use of alcohol			0.59
Yes	269 (93.7)	198 (92.5)	
No	18 (6.3)	16 (7.5)	
Employment status			0.24
Yes	260 (90.6)	200 (93.5)	
No	27 (9.4)	14 (6.5)	
Stage			0.82
Stage 2–3	145 (50.5)	106 (49.5)	
Stage 4	142 (49.5)	108 (50.5)	
BMI			
<21	58 (20.8)	64 (29.9)	0.01
≥21	229 (79.8)	150 (70.1)	
Time since cancer diagnosis			
<6	173 (60.3)	109 (50.9)	0.03
≥6	114 (39.7)	105 (49.1)	
Number of comorbidities			
0–1	228 (79.7)	169 (79.3)	0.91
≥2	58 (20.3)	44 (20.7)	
Cancer type			
Gastrointestinal cancer	137(47.7)	88(41.1)	0.14
Non-gastrointestinal cancer	150(52.3)	126(58.9)	
Stage			
2–3	145(50.5)	106(49.5)	0.24
4	142(49.5)	108(50.5)	
Pain score			
Median ± SD	1,14 + 1.36	1.79 + 1.79	0.00
İnsomnia score			
0–7	191 (66.6)	120 (56.1)	0.01
≥8	96 (33.4)	94 (43.9)	

*p-values of <0.05 were considered significant and shown in bold. Pain score range: 0 (best)–10 (worst).

To identify independent predictors of depression, a multivariable logistic regression analysis was performed, including variables found to be significant in the univariate analysis. Being female (OR: 1.481, 95% CI: 1.011–2.168, *p* = 0.04), BMI ≥ 21 (OR: 1.665, 95% CI: 1.081–2.564, *p* = 0.02), higher pain scores (OR: 1.269, 95% CI: 1.122–1.436, *p* < 0.001), insomnia (OR: 1.626, 95% CI: 1.109–2.384, *p* = 0.01), and weak social support (OR: 2.004, 95% CI: 1.046–3.839, *p* = 0.03) were identified as independent associated factors for high depressive symptoms. In contrast, treatment duration ≥6 months was not found to be independently associated with depression in the multivariate model (*p* = 0.26) (Table 3).

**Table 3:** Multivariable logistic regression analysis of variables significant in univariate analysis.

Variables	*p*-value*	Odds ratio (OR)	95% CI
Pain score	*p* < 0.001	1.269	1.122–1.436
Sex	0.04	1.481	1.011–2.168
BMI	0.02	1.665	1.081–2.564
Time since cancer diagnosis	0.26	1.242	0.850–1.815
İnsomnia score	0.01	1.626	1.109–2.384
Social support	0.03	2.004	1.046–3.839

*p-values of <0.05 were considered significant.

## Discussion

With improvements in healthcare systems, the proportion of the older adult population has been steadily increasing, resulting in geriatric patients constituting a significant majority of cancer cases (14). Geriatric patients are particularly prone to psychological stress due to the physical challenges of cancer treatment, as well as accompanying comorbidities, loss of spouses and friends, and reduced social support (2). In our study, we evaluated the prevalence of depressive symptoms and associated factors in geriatric patients aged 65 years and older receiving chemotherapy for cancer. We identified significant associations between a high depression score and being female, weak social support, BMI ≥ 21, treatment duration ≥6 months, and higher pain scores.

According to a 2021 review by Obuobi-Donkor et al., the prevalence of major depressive disorder in geriatric populations varies widely, ranging from 5.37–56%, depending on factors such as assessment tools, diagnostic criteria, and patient characteristics (15). Studies reporting lower prevalence rates often utilized clinical diagnostic interviews in community-dwelling older adults without major comorbidities. In contrast, higher prevalence rates were observed in studies involving patients with chronic illnesses or institutionalized populations, particularly when using self-reported screening instruments. Our study, which found a 42.7% prevalence of high depressive symptoms using the GDS in geriatric cancer patients actively receiving chemotherapy, aligns with the upper end of this range. This elevated rate is likely due to the combined burden of cancer diagnosis, chemotherapy side effects, reduced social support, and multiple comorbidities, factors similar to those observed in studies reporting higher prevalence. It is well known that the psychological stress associated with a cancer diagnosis and its treatment further increases the risk of depression (2, 3).

The timing of depression assessment is a critical factor influencing prevalence estimates. In our study, depressive symptoms were specifically evaluated during the chemotherapy treatment phase, a period associated with heightened physical burden and psychological stress. This timing likely contributed to the relatively high prevalence (42.7%) of depressive symptoms observed in our geriatric cohort. Previous studies support the influence of timing on depression rates. For example, Zhao et al. reported higher depression prevalence among cancer survivors who were within 1 year of diagnosis or still receiving active treatment (16). Similarly, a recent umbrella review found the global prevalence of depression among cancer survivors to be 33.16%, with rates increasing to 43.25% during the COVID-19 pandemic, especially in patients undergoing treatment (17). In line with this, a study conducted at Soroka University Medical Center (SUMC) in Israel evaluated depression during active oncologic treatment and reported a lower prevalence of 22.6% using the PROMIS Emotional Distress–Depression scale in a broader adult cancer population (18). Additionally, a general review on geriatric mental health highlighted that depression prevalence in older adults can range from 5.37 to 56%, with rates significantly influenced by the timing of assessment, comorbid conditions, and social factors such as isolation (15). Collectively, these findings underscore that assessing depression during chemotherapy offers important insights into the peak psychological vulnerability of older adult cancer patients and highlights the need for timely mental health interventions in this population.

In a 2016 study, Sassarini reported that women are more susceptible to depression than men due to hormonal changes, particularly fluctuations in estrogen levels. Similarly, Albert et al. demonstrated comparable findings in their 2019 study (19, 20). According to studies by Judd et al. (21) and Lin et al. (22), hormonal changes associated with menopause further increase the prevalence of depressive symptoms in the geriatric period. A 2021 review by Maier et al. found that being female was a significant factor contributing to depression in patients aged 65 years and older (23). Moreover, a 2003 meta-analysis by Cole et al. identified being female as an independent associated factor for depression in older adult patients (24). In alignment with the literature, our study also found that being female was associated with higher depressive symptom scores. In addition to biological factors such as hormonal changes, cross-cultural studies suggest that differences in social roles, such as increased caregiver responsibilities, financial dependency, and social isolation, may also contribute to higher rates of depression among older adult women (23). Although our study did not directly assess these role-related factors, future studies may benefit from incorporating structured evaluations of caregiving burden, economic dependence, and social role stress. Including such variables could provide a more comprehensive understanding of the gender-specific pathways contributing to depression in older adults.

According to a 2022 review by Bottaro et al., there is a strong correlation between receiving robust social support and effective coping mechanisms in cancer patients, regardless of cancer type (25). Geriatric cancer patients require social support and care not only to improve nutrition, ensure regular treatment attendance, and monitor physical side effects more effectively, but also to prevent feelings of loneliness by fostering a sense of belonging within the family. In Middle Eastern countries, this support is typically provided effectively by family members, and studies have shown that patients with strong social support experience fewer depressive symptoms (26–29). In our study, geriatric patients living with their families were classified as receiving strong social support due to the high-quality care and social connections they received. In contrast, patients living alone, with a caregiver, or in nursing homes were classified as having weak social support. We found that weak social support was associated with higher depressive symptom scores. Similar to our findings, other studies have demonstrated that strong social support during cancer treatment not only improves treatment adherence but also reduces psychological stress (30, 31).

In our study, a treatment duration of 6 months or longer was found to be significantly associated with higher depressive symptom scores in the univariate analysis. This may be due to prolonged exposure to the physical effects of chemotherapy, increased psychological fatigue, extended social isolation, and financial burden—all of which can contribute to emotional distress. However, this association did not remain significant in the multivariable regression model, possibly because its effect was mediated by other variables such as pain or reduced social support, which were independently associated with depression. This finding suggests that treatment duration may not directly influence depression, but rather exerts its impact through related psychosocial and clinical factors.

It is well known that the prevalence of chronic pain is high in geriatric patients (32, 33). Among cancer patients, this rate is significantly higher due to cancer-related pain (34). According to a 2017 study by Zis et al., chronic pain can exacerbate depression, while depression can, in turn, amplify the perception of pain, indicating a bidirectional relationship between the two. Similarly, a study conducted by Sheng et al. in the same year reported comparable findings (35, 36). Therefore, the diagnosis and management of both chronic pain and depression are crucial in patient care. In our study, patients with higher pain scores were found to have higher depressive symptom scores, consistent with the literature. Notably, the multivariable analysis revealed that, for each 1-point increase in pain score, the odds of experiencing depressive symptoms increased by approximately 27%. This finding highlights the strong clinical relevance of adequate pain control in improving the psychological well-being of geriatric cancer patients.

According to a 2018 meta-analysis by Gebara et al., similar to chronic pain, there is a bidirectional relationship between insomnia and depression. Insomnia can lead to depression, and depression can, in turn, cause insomnia (37). A 2021 study by Tsaras et al. further supports this bidirectional relationship in geriatric patients (38). Therefore, the diagnosis and management of both insomnia and depression are crucial in geriatric patients. In our study, we used the ISI in conjunction with the GDS to assess insomnia. Consistent with the literature, we found that patients with insomnia had higher depressive symptom scores (13).

In a 2019 study conducted in the United Kingdom involving 215,125 participants, Mulugeta et al. examined the relationship between depression and obesity and found a genetic predisposition to higher BMI in individuals with depression (39). Similarly, a 2019 study by Tyrrell et al. identified an increased susceptibility to depression in individuals with a high BMI (40). Consistent with the literature, our study found that patients with a higher BMI had higher depressive symptom scores.

In this study, we found no significant association between depressive symptoms and ECOG performance status, education level, cancer type, or disease stage. Although these null findings may suggest a limited role of these factors in this context, they should be interpreted with caution. The relatively homogeneous distribution of ECOG scores (with the majority of patients having an ECOG score of 1–2) and the limited variability in educational background may have reduced the statistical power to detect significant associations. Moreover, while our data did not demonstrate a link between cancer type or stage and depression, previous research suggests that certain malignancies may exert a stronger psychological impact (41, 42). Therefore, the absence of significant findings in our study does not preclude a possible relationship, which may become evident in larger or more stratified samples.

It is also important to emphasize that the GDS is a screening tool rather than a diagnostic instrument. Although it is validated and widely used in geriatric populations, it does not replace structured clinical interviews based on standardized diagnostic criteria such as the DSM-5. Therefore, the prevalence of high depressive symptom scores reported in our study may not directly reflect the actual prevalence of major depressive disorder. This distinction should be taken into account when interpreting the findings.

Our study has several limitations. First, it was conducted in a single center, which may limit the generalizability of the findings. The results might differ in studies involving larger and more heterogeneous populations across various geographic and cultural contexts. In particular, the traditional family structure in Türkiye, where older adults often live with family members and receive informal caregiving, may differ from those in other regions. This cultural difference could have influenced the strength of the observed association between social support and depression, and caution is warranted when extrapolating these results to populations with different social dynamics. Second, the cross-sectional design of the study prevents causal inferences regarding the relationship between depression and associated factors. Longitudinal studies are needed to explore these associations over time. Third, although excluding patients with cognitive impairment allowed for more accurate self-reporting, it may have introduced selection bias and reduced the generalizability of the findings, particularly among the most vulnerable patients. Fourth, although the GDS and ISI are validated screening tools frequently used in geriatric research, the absence of structured psychiatric interviews based on formal diagnostic criteria, such as the *Diagnostic and Statistical Manual of Mental Disorders, Fifth Edition* (DSM-5), may limit diagnostic precision. Fifth, while all patients completed the GDS and ISI without missing data, we did not specify how missing data were handled for other variables, which may affect transparency. Sixth, the classification of social support was based solely on living arrangements, which may oversimplify the multifaceted nature of psychosocial support. Finally, BMI was analyzed categorically using a threshold recommended by geriatric oncology guidelines; however, modeling BMI as a continuous variable may provide more detailed insights and should be considered in future studies.

## Conclusion

Our study highlights the high prevalence of depressive symptoms among geriatric cancer patients, demonstrating associations with factors such as being female, high BMI, weak social support, prolonged treatment duration, and coexisting conditions, including insomnia and chronic pain. Early diagnosis and management of depression in this population are crucial for enhancing quality of life and improving treatment adherence. Strengthening social support mechanisms and effectively managing associated conditions such as pain and insomnia may help reduce the risk of depression. However, further research is needed to explore the underlying mechanisms and to develop targeted, multidisciplinary strategies for preventing and managing depression in geriatric cancer patients. Future studies should focus on multicenter longitudinal designs to better clarify causal relationships and examine additional variables, such as chemotherapy drug types, treatment phase, and gender-specific risk profiles, which may contribute to more personalized and effective interventions.

## Data Availability

The raw data supporting the conclusions of this article will be made available by the authors, without undue reservation.

## References

[1] Diaz FC Hamparsumian A Loh KP Verduzco-Aguirre H Abdallah M Williams GR. Geriatric oncology: a 5-year strategic plan. Am Soc Clin Oncol Educ Book. (2024) 44:e100044. doi: 10.1200/EDBK_100044, PMID: 38709980 PMC11463154

[2] Saracino RM Nelson CJ. Identification and treatment of depressive disorders in older adults with cancer. J Geriatric Oncol. (2019) 10:680–4. doi: 10.1016/j.jgo.2019.02.005, PMID: 30797709 PMC7457378

[3] Beauplet B Soulie O Niemier J-Y Pons-Peyneau C Belhadi D Couffignal C. Dealing with the lack of evidence to treat depression in older patients with cancer: French societies of geriatric oncology (SOFOG) and PsychoOncology (SFFPO) position paper based on a systematic review. Support Care Cancer. (2021) 29:563–71. doi: 10.1007/s00520-020-05682-9, PMID: 32870413

[4] Weiss Wiesel TR Nelson CJ Tew WP Hardt M Mohile SG Owusu C. The relationship between age, anxiety, and depression in older adults with cancer. Psycho-Oncology. (2015) 24:712–7. doi: 10.1002/pon.3638, PMID: 25099337 PMC4320028

[5] Extermann M Hurria A. Comprehensive geriatric assessment for older patients with cancer. J Clin Oncol. (2007) 25:1824–31. doi: 10.1200/JCO.2007.10.6559, PMID: 17488980

[6] Yesavage JA Brink TL Rose TL Lum O Huang V Adey M. Development and validation of a geriatric depression screening scale: a preliminary report. J Psychiatr Res. (1982) 17:37–49. doi: 10.1016/0022-3956(82)90033-4, PMID: 7183759

[7] Ertan T Eker E. Reliability, validity, and factor structure of the geriatric depression scale in Turkish elderly: are there different factor structures for different cultures? Int Psychogeriatr. (2000) 12:163–72. doi: 10.1017/S1041610200006293, PMID: 10937537

[8] Durmaz B Soysal P Ellidokuz H Isik AT. Validity and reliability of geriatric depression scale-15 (short form) in Turkish older adults. North Clin Istanb. (2018) 5:216–20. doi: 10.14744/nci.2017.85047, PMID: 30688929 PMC6323561

[9] Burke WJ Roccaforte WH Wengel SP. The short form of the geriatric depression scale: a comparison with the 30-item form. J Geriatr Psychiatry Neurol. (1991) 4:173–8. doi: 10.1177/089198879100400310, PMID: 1953971

[10] Mohile SG Dale W Somerfield MR Schonberg MA Boyd CM Burhenn PS. Practical assessment and management of vulnerabilities in older patients receiving chemotherapy: ASCO guideline for geriatric oncology. J Clin Oncol. (2018) 36:2326–47. doi: 10.1200/JCO.2018.78.8687, PMID: 29782209 PMC6063790

[11] Jan A Weir CB. BMI classification percentile and cut off points. StatPearls: Treasure Island, FL, USA (2021) 1–4. 31082114

[12] Karcioglu O Topacoglu H Dikme O Dikme O. A systematic review of the pain scales in adults: which to use? Am J Emerg Med. (2018) 36:707–14. doi: 10.1016/j.ajem.2018.01.008, PMID: 29321111

[13] Morin CM Belleville G Bélanger L Ivers H. The insomnia severity index: psychometric indicators to detect insomnia cases and evaluate treatment response. Sleep. (2011) 34:601–8. doi: 10.1093/sleep/34.5.601, PMID: 21532953 PMC3079939

[14] Okoli GN Stirling M Racovitan F Lam OLT Reddy VK Copstein L. Integration of geriatric assessment into clinical oncology practice: a scoping review. Curr Probl Cancer. (2021) 45:100699. doi: 10.1016/j.currproblcancer.2020.100699, PMID: 33468334

[15] Obuobi-Donkor G Nkire N Agyapong VI. Prevalence of major depressive disorder and correlates of thoughts of death, suicidal behaviour, and death by suicide in the geriatric population—a general review of literature. Behav Sci. (2021) 11:142. doi: 10.3390/bs11110142, PMID: 34821603 PMC8614881

[16] Zhao G Okoro CA Li J White A Dhingra S Li C. Current depression among adult cancer survivors: findings from the 2010 behavioral risk factor surveillance system. Cancer Epidemiol. (2014) 38:757–64. doi: 10.1016/j.canep.2014.10.002, PMID: 25455653 PMC11000226

[17] Getie A Ayalneh M Bimerew M. Global prevalence and determinant factors of pain, depression, and anxiety among cancer patients: an umbrella review of systematic reviews and meta-analyses. BMC Psychiatry. (2025) 25:156. doi: 10.1186/s12888-025-06599-5, PMID: 39972435 PMC11841195

[18] Shalata W Gothelf I Bernstine T Michlin R Tourkey L Shalata S. Mental health challenges in Cancer patients: a cross-sectional analysis of depression and anxiety. Cancers. (2024) 16:2827. doi: 10.3390/cancers16162827, PMID: 39199598 PMC11352929

[19] Sassarini J. Depression in midlife women. Maturitas. (2016) 94:149–54. doi: 10.1016/j.maturitas.2016.09.004, PMID: 27823736

[20] Albert KM Newhouse PA. Estrogen, stress, and depression: cognitive and biological interactions. Annu Rev Clin Psychol. (2019) 15:399–423. doi: 10.1146/annurev-clinpsy-050718-095557, PMID: 30786242 PMC9673602

[21] Judd FK Hickey M Bryant C. Depression and midlife: are we overpathologising the menopause? J Affect Disord. (2012) 136:199–211. doi: 10.1016/j.jad.2010.12.010, PMID: 21269707

[22] Lin H Hsiao M Liu Y Chang C. Perimenopause and incidence of depression in midlife women: a population-based study in Taiwan. Climacteric. (2013) 16:381–6. doi: 10.3109/13697137.2012.707706, PMID: 22963154

[23] Maier A Riedel-Heller SG Pabst A Luppa M. Risk factors and protective factors of depression in older people 65+ a systematic review. PLoS One. (2021) 16:e0251326. doi: 10.1371/journal.pone.0251326, PMID: 33983995 PMC8118343

[24] Cole MG Dendukuri N. Risk factors for depression among elderly community subjects: a systematic review and meta-analysis. Am J Psychiatry. (2003) 160:1147–56. doi: 10.1176/appi.ajp.160.6.1147, PMID: 12777274

[25] Bottaro R Craparo G Faraci P. What is the direction of the association between social support and coping in cancer patients? A systematic review. J Health Psychol. (2023) 28:524–40. doi: 10.1177/13591053221131180, PMID: 36314888

[26] Şahin E Topkaya N Gençoğlu C Ersanlı E. Prevalence and correlates of hopelessness among Turkish elderly people living with family or in nursing homes. Societies. (2018) 8:39. doi: 10.3390/soc8020039

[27] Sanlier N Yabanci N. Mini nutritional assessment in the elderly: living alone, with family and nursing home in Turkey. Nutr Food Sci. (2006) 36:50–8. doi: 10.1108/00346650610642197

[28] Al-Krenawi A Graham JR. Culturally sensitive social work practice with Arab clients in mental health settings. Health Soc Work. (2000) 25:9–22. doi: 10.1093/hsw/25.1.9, PMID: 10689599

[29] Vahedparast H Mohammadi E Ahmadi F Farhadi A. The role of social support in adherence to treatment regimens: experiences of patients with chronic diseases. Medical-surgical. Nurs J. (2018) 7:e69646. doi: 10.5812/msnj.69646, PMID: 40365301

[30] Usta YY. Importance of social support in cancer patients. Asian Pac J Cancer Prev. (2012) 13:3569–72. doi: 10.7314/APJCP.2012.13.8.3569, PMID: 23098436

[31] Hermann M Goerling U Hearing C Mehnert-Theuerkauf A Hornemann B Hövel P. Social support, depression and anxiety in Cancer patient-relative dyads in early survivorship: an actor-partner interdependence modeling approach. Psycho-Oncology. (2024) 33:e70038. doi: 10.1002/pon.70038, PMID: 39643936 PMC11624292

[32] Tinnirello A Mazzoleni S Santi C. Chronic pain in the elderly: mechanisms and distinctive features. Biomol Ther. (2021) 11:1256. doi: 10.3390/biom11081256, PMID: 34439922 PMC8391112

[33] Borsheski R Johnson QL. Pain management in the geriatric population. Mo Med. (2014) 111:508–11. PMID: 25665235 PMC6173536

[34] Finnerty D O’Gara Á Buggy DJ. Managing pain in the older cancer patient. Curr Oncol Rep. (2019) 21:1–10. doi: 10.1007/s11912-019-0854-7, PMID: 31728653

[35] Zis P Daskalaki A Bountouni I Sykioti P Varrassi G Paladini A. Depression and chronic pain in the elderly: links and management challenges. Clin Interv Aging. (2017) 12:709–20. doi: 10.2147/CIA.S113576, PMID: 28461745 PMC5407450

[36] Sheng J Liu S Wang Y Cui R Zhang X. The link between depression and chronic pain: neural mechanisms in the brain. Neural Plast. (2017) 2017:1–10. doi: 10.1155/2017/9724371, PMID: 28706741 PMC5494581

[37] Gebara MA Siripong N DiNapoli EA Maree R Germain A Reynolds C. Effect of insomnia treatments on depression: a systematic review and meta-analysis. Depress Anxiety. (2018) 35:717–31. doi: 10.1002/da.22776, PMID: 29782076

[38] Tsaras K Tsiantoula M Papathanasiou IV Papagiannis D Chatzi M Fradelos EC. Predictors of depression and insomnia in community-dwelling elderly people: a cross-sectional evidence of their bidirectional relationship. Cureus. (2021) 13:e13965. doi: 10.7759/cureus.13965 33880299 PMC8052590

[39] Mulugeta A Zhou A Vimaleswaran KS Dickson C Hyppönen E. Depression increases the genetic susceptibility to high body mass index: evidence from UK biobank. Depress Anxiety. (2019) 36:1154–62. doi: 10.1002/da.22963, PMID: 31609059

[40] Tyrrell J Mulugeta A Wood AR Zhou A Beaumont RN Tuke MA. Using genetics to understand the causal influence of higher BMI on depression. Int J Epidemiol. (2019) 48:834–48. doi: 10.1093/ije/dyy223, PMID: 30423117 PMC6659462

[41] Yildirim D. Relationship between the depression levels and nutritional statuses of advanced stage cancer patients. Palliat Support Care. (2022) 20:654–61. doi: 10.1017/S1478951521001516, PMID: 34588082

[42] Linden W Vodermaier A MacKenzie R Greig D. Anxiety and depression after cancer diagnosis: prevalence rates by cancer type, gender, and age. J Affect Disord. (2012) 141:343–51. doi: 10.1016/j.jad.2012.03.025, PMID: 22727334

